# IS SUPERFICIAL COLORECTAL LESIONS WITH LOW AND HIGH GRADES INTRAEPITHELIAL NEOPLASMS MORE PREVALENT IN OLDER ABOVE 65 YEARS?

**DOI:** 10.1590/0102-672020190001e1478

**Published:** 2019-12-20

**Authors:** Nildete Rodrigues DIGER, Luiz Fernando KUBRUSLY, Paulo Afonso Nunes NASSIF, Artur Adolfo PARADA, Giovana Tonello BOLSI, Harymy Costa Barros TEIXEIRA, Osvaldo MALAFAIA

**Affiliations:** 1 Postgraduate Program in Principles of Surgery, Mackenzie Evangelical School of Medicine - Paraná, Curitiba, PR, Brazil; 2 Digestive Endoscopy Service, 9 de Julho Hospital, São Paulo, SP, Brazil

**Keywords:** Colonoscopy, Aged, Colorectal neoplasms, Carcinoma in situ, Colonoscopia, Idoso, Neoplasias colorretais, Carcinoma in situ

## Abstract

**Background:**

Colorectal cancer has a higher incidence in the rectum and sigmoid. However, with the expansion of the diagnosis of superficial lesions interest in the diagnosis and in the role they play in colorectal carcinogenesis has increased.

**Aim:**

To verify the behavior of superficial lesions of the colon and rectum, comparing the pathological and endoscopic findings, below and above 65 years.

**Methods:**

Cross-sectional study with prospective evaluation of standard protocol, where 200 patients with colorectal superficial lesions were evaluated; they were submitted to colonoscopy and mucosectomy of these lesions. They were divided in two age groups, below and above 65 years.

**Results:**

One hundred-and-eight were women (54%) and 92 men (46%). Most colon lesions were localized in the right colon (95%) and the remaining (5%) in the rectum. In endoscopy, 77.20% were granular lesions in patients under 65 years and 77.90% above. Colon histology showed low grade intraepithelial neoplasia, being 69.79% in patients under and 73.70% in above 65 years. In rectum, above 65 years the incidence of high-grade intraepithelial neoplasia was higher (66.70%).

**Conclusion:**

The superficial colorectal lesions have been more endoscopically diagnosed today, and the highest incidence is the granular type, both in the colon and rectum, regardless of age. Regardless the age, histologically colon lesions were more as low grade intraepithelial neoplasia. In rectum, there was distinction for both age groups, being more frequent high grade intraepithelial neoplasia in patients over 65 years.

## RESUMO

**Racional:**

O câncer colorretal tem maior incidência no reto e sigmoide. Porém, com a ampliação do diagnóstico das lesões superficiais do cólon e reto tem-se aumentado o interesse por elas no diagnóstico e no papel que elas representam na carcinogênese colorretal.

**Objetivo:**

Verificar o comportamento das lesões superficiais do cólon e reto, comparando os achados anatomopatológicos com os endoscópicos em duas faixas etárias, abaixo e acima de 65 anos.

**Métodos:**

Estudo retrospectivo transversal onde foram avaliados 200 pacientes com lesões superficiais colorretais submetidos à colonoscopia e mucosectomia destas lesões.

**Resultados:**

Foram 108 mulheres (54%) e 92 homens (46%). A maioria das lesões localizou-se no cólon direito (95%) e as demais (5%) no reto. Quanto ao aspecto endoscópico 77,20% tinham superfície granulosa para pacientes abaixo de 65 anos e 77,90% para os acima. Quanto ao aspecto histológico no cólon a maioria, independentemente da idade, mostrou ser neoplasia intraepitelial de baixo grau, enquanto que no reto, nos mais idosos, a incidência de neoplasia intraepitelial de alto grau foi maior (66,70%).

**Conclusão:**

As lesões superficiais colorretais têm sido mais diagnosticadas pela colonoscopia e a forma granular apresenta maior incidência, tanto nos mais jovens como nos mais idosos. Os achados anatomopatológicos no cólon, independente da faixa etária, foram mais de neoplasia intraepitelial de baixo grau. No reto observou-se distinção para as duas faixas etárias, sendo mais frequentes os casos de neoplasia intraepitelial de alto grau para os pacientes acima de 65 anos.

## INTRODUCTION

Colorectal malignant neoplasia is considered the third leading cause of cancer in the world and the second in death rate from cancer in North America and Western Europe^15^. In Brazil, the incidence varies according to the geographic region, being higher in the South and Southeast and lower in the Midwest, Northeast and North^5^ ^,^ ^6^. Currently, it represents the second most common cancer diagnosed in women and the third in men^5^ ^,^ ^22^.

Colorectal cancer has a higher incidence in the rectum and sigmoid^8^. However, nowadays, with the expansion of the diagnosis of non-polypoid lesions - which are superficial lesions of the colon and rectum - the interest for them in the diagnosis and the role they play in colorectal carcinogenesis has increased^2^.

Superficial lesions are often flat or slightly elevated and some have lateral growth4. Colonoscopy has been used as a screening, diagnosis and treatment method^9,22^ and represents the only means that can reduce the incidence of colorectal cancer allowing lesions resections^14^ ^,^ ^18^. The detection of superficial lesion in asymptomatic patients undergoing colonoscopy is frequent and varies between 10-60%^17^.

Superficial lesions can be difficult to diagnose; but, experienced endoscopists using current techniques - image magnification and chromoscopy - can often do it, evaluating them anatomopathologically. The most widely used pathological classification is the revised Vienna^4^ classification that uses epithelial changes, their propagation and/or invasion of the submucosa. However, in endoscopic vision the most common classification is Paris, dividing the lesions into superficially elevated, flat, depressed lesions and those presenting with horizontal growth^5^.

Superficial lesions that are usually flat or slightly elevated tend to spread laterally, whereas in depressed lesions, growth progresses deep into the colon wall, thus increasing submucosa (sm1) invasion even in minor lesions^13^.

Lateral spreading lesions are generally defined as surface areas equal to or larger than 10 mm in diameter, which exhibit significant horizon lateral growth in the colon wall in relation to polypoid or vertical growth^3^. They and larger polyps have an increased frequency of dysplasia and greater local invasion when compared to pedicle lesions of the same size^5^ ^,^ ^20^. The lateral spreading, according to the endoscopic aspect, are divided into two types: granular and non-granular; on the other hand, these types have two subtypes: homogeneous or nodular granular lateral spreading lesions, and non-granular lateral spreading lesions, elevated/plane or areas of depression or pseudodepression^7^ ^,^ ^10^ ^,^ ^14^.

Granular lesions of the homogeneous subtype have a low risk (less than 2%) of invading the submucosa (sm1) regardless of its size, whereas nodular granular risk rises to 7.1% for lesions smaller than 20 mm and to 38% equal to or greater than 30 mm^21^. Regarding non-granular lesions, the risk of submucosal invasion is higher, especially those with pseudodepression, which show 12.5% when smaller than 20 mm and 83.3% when greater than 30 mm^12^.

Granular lesions are responsible for 60-80% of cases, non-granular for 20-40% and depressed for 1-6% of total colorectal surfaces^1^ ^,^ ^11^.

This study aimed to verify the behavior of superficial lesions of the colon and rectum, comparing the pathological findings with the endoscopic findings in two age groups, under and over 65 years.

## METHODS

This study was approved by the Research Ethics Committee of the Evangelical School of Paraná, Curitiba, PR under no. 3,400,247. This is an observational, retrospective and cross-sectional study of standard protocols of patients with colorectal superficial lesions who underwent endoscopic resection (mucosectomy) over a period of four years (February 2010 to December 2014) at the Digestive Endoscopy Service of Hospital 9 de July, São Paulo, SP, Brazil. Were included 200 patients referred for colonoscopic mucosectomy. The age range considered was above and below 65 years, regardless of gender.

The exams were performed after proper preparation of the colon with a light diet without residues the day before and with 20% mannitol on the day of the exam. All were in good clinical condition, with no contraindication for colonoscopy and the associated procedure. The exams were performed with sedation and anesthetic follow-up, without any complications.

The devices used were Olympus, Pentax and Fujinon and the materials for mucosectomy were: diathermic loop, hemostasis metal clips, injector catheter and, for the elevation of the lesions, 1: 10000 dilution adrenaline solution and saline (0, 9%).

After performing the mucosectomies, the specimens were submitted and evaluated by a single pathologist.

Regarding the histopathological pattern, the lesions were ordered by the Vienna Classification^4^, which classifies them into categories: 1 (negative for neoplasia); 2 (undefined for cancer); 3 (low grade intraepithelial neoplasia); 4 (high grade intraepithelial neoplasia); and 5 (neoplasm with submucosal invasion). Regarding the endoscopic pattern, the lesions followed the Paris classification which considers them as elevated (O-IIa), flat (O-IIb), depressed (O-IIc), excavated (O-III) and the laterally growing type spread or lateral spreading injury (LST).

**FIGURE 1:**
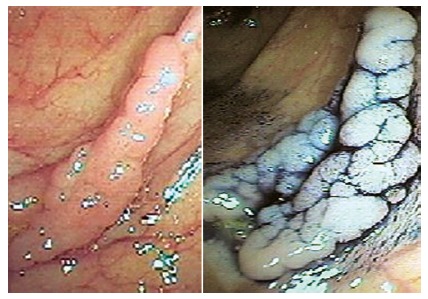
Lateral spreading lesion

### Statistical analysis

It was performed using the MS-Excel spreadsheet and the IBPSPSS statistical package. To compare age groups and lesion size, the likelihood ratio test was applied to verify possible differences between the two control variables: age and lesion size. A significance level of 5% (p=0.05) was adopted.

## RESULTS

**FIGURE 2:**
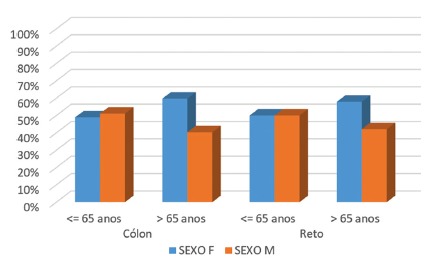
Distribution of patients by age and gender

Of the 200 patients included, under and over 65, 108 were women (54%) and 92 men (46%). Of the regions analyzed - colon and rectum - in women it was found that, under 65 years, 49% were in the colon and 50% in the rectum; men in this same age group were 51% in the colon and 50% in the rectum; for women over 65, 60% were in the colon and 42% in the rectum, and in men 40% were in the colon and 42% in the rectum (Figure 2).

Most lesions of these two age groups were more frequently located in the right colon, especially in the ascending segment. It was 50% for patients under 65 years and 45% for those above; the other locations are shown in Table 1.

**TABLE 1:** Location of colon and rectum lesions

Site	Age range (years)	Location				
		Ascending	Cecum	Descending	Rectum	Sigmoid
Colon	<=65	50.00%	16.70%	24.40%	0.00%	8.90%
	>65	45.50%	19.50%	19.50%	0.00%	15.60%
	Total	47.90%	18.00%	22.20%	0.00%	12.00%
Rectum	<=65	0.00%	0.00%	0.00%	100.00%	0.00%
	>65	0.00%	0.00%	0.00%	100.00%	0.00%
	Total	0.00%	0.00%	0.00%	100.00%	0.00%

**FIGURE 3:**
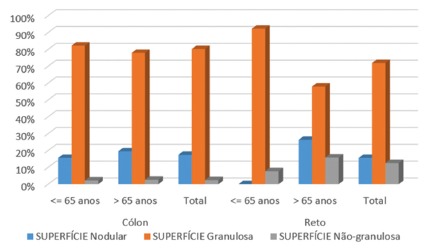
Endoscopic aspects of the lesions

Regarding the endoscopic aspect of the lesions, it was observed that most of them had granular surface, being 77.20% in patients under 65 years and 77.90% above. In the above, the nodular granular aspect was verified in 19.50%, and in the younger in 15.60% (Figure 3).

In the colonic lesions, in both age groups, most were low grade intraepithelial neoplasia, being 69.70% under 65 years and 73.70% above. In the rectum, there was a higher incidence of high grade intraepithelial neoplasia in the upper range in 66.70% and 42.90% in the below (Table 2).

**TABLE 2:** Histopathological pattern of colon and rectum lesions

Site	Age range (years)	Anatomopathology		
		NIE high grade	NIE low grade	Hyperplastic polyp
Colon	<=65	21%	70%	9%
	>65	20%	74%	7%
	Total	21%	72%	8%
Rectum	<=65	43%	50%	7%
	>65	67%	28%	6%
	Total	56%	37%	6%

NIE=intraepithelial neoplasia

**FIGURE 4:**
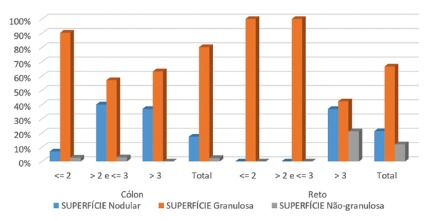
Endoscopic aspect of the lesion according to colon and rectum size and location

Regarding size, the highest frequency of lesions ranged from 2 to 3 cm in both colon and rectum. A large percentage of lesions larger than 3 cm were also found, 52.60% of them in the colon and 55% in the rectum. Most lesions according to the endoscopic aspect were granular lesions and more common in the ascending colon (Figure 4).

Regarding the anatomopathological outcome, most in the colon regardless of size, even larger than 3 cm, was classified as low grade intraepithelial neoplasia. However, in the rectum, the lesions were different, with the majority presenting as high-grade intraepithelial neoplasia, 66.70% for 2 to 3 cm lesions, and 63.20% for lesions greater than 3 cm (Table 3). 

**TABLE 3:** Anatomopathological outcome of lesions according to size and location in colon and rectum

Site	Size (cm)	Anatomopathology		
		NIE high grade	NIE low grade	Hyperplastic polyp
Colon	<=2	14%	75%	11%
	> 2 and <=3	35%	65%	0%
	>3	32%	63%	5%
	Total	21%	72%	8%
Rectum	<=2	40%	40%	20%
	> 2 and <=3	67%	33%	0%
	>3	63%	37%	0%
	Total	56%	38%	6%

NIE=intraepithelial neoplasia

## DISCUSSION

Colorectal cancer is one of the most common cancers worldwide, and colonoscopy is the gold standard for detecting precancerous lesions at risk of progression to colorectal neoplasia^5^ ^,^ ^19^.

In recent times there has been greater interest in superficial non-polypoid colorectal lesions, which are present in about 10-60% of colonoscopies performed on asymptomatic patients^17^.

These lesions are superficially elevated, flat, depressed, hollow, and superficially growing colorectal tumors and are considered colorectal precancerous lesions^5^.

This study demonstrated that they have distinct location, endoscopic, pathological features, malignancy potential and invasion. They are often difficult to diagnose, but with experienced endoscopists and the current endoscopic arsenal, such as imaging magnification techniques, more and more lesions are diagnosed^14^.

It was observed in the literature^14^ that the prevalence of superficial lesions is more common in the right colon, regardless of age and lesion size.

Among the superficial non-polypoid lesions, there is a subgroup that has been highlighted in the current panorama, which are the lateral spreading, which grow in the lateral horizontal direction in the colon wall^16^.

Most superficial lesions are known to have endoscopy with granular appearance, and thus with lower potential for malignancy in relation to the non-granular pattern^12^.

Recent studies^10,14^ have indicated that superficial lesions with lateral spreading represent 17.2% of advanced colorectal neoplasms and that they may develop high grade intraepithelial neoplasms with incidence rates ranging from 20.9% to 33.8%. They may also progress to deeper cancer with invasion of the submucosal layer as was also observed in this study.

It has been found here that there are important differences between superficial lesions in the colon and rectum related to age. Although the histological pattern is not different in both age groups, when the lesions were located in the colon, both groups presented histological pattern of low grade intraepithelial neoplasia; in the rectum, this pattern had a totally different appearance, with 66.70% of the cases over 65 years old with high grade intraepithelial neoplasia. It was also observed that the larger the lesion, from 2 cm or rectum, also increases the degree of high grade intraepithelial neoplasia, totaling 66.70% for lesions of 2 to 3 cm and 63.20% in larger than 3 cm. These findings are also cited in the literature^7^ ^,^ ^12^.

## CONCLUSION

Colorectal superficial lesions have been more diagnosed by colonoscopy and the granular form has a higher incidence, both in younger and older. The anatomopathological findings in the colon, regardless of age, were more of low grade intraepithelial neoplasia. In the rectum it was observed that there is a distinction for the two age groups, being the most frequent cases of high grade intraepithelial neoplasia for patients over 65 years.
